# Sirtuins at the crossroads of stemness, aging, and cancer

**DOI:** 10.1111/acel.12685

**Published:** 2017-10-10

**Authors:** Carol O'Callaghan, Athanassios Vassilopoulos

**Affiliations:** ^1^ Laboratory for Molecular Cancer Biology Department of Radiation Oncology Feinberg School of Medicine Northwestern University Chicago IL USA; ^2^ Robert H. Lurie Comprehensive Cancer Center Northwestern University Chicago IL USA

**Keywords:** Sirtuins, stem cells, aging, cancer, EMT, calorie restriction

## Abstract

Sirtuins are stress‐responsive proteins that direct various post‐translational modifications (PTMs) and as a result, are considered to be master regulators of several cellular processes. They are known to both extend lifespan and regulate spontaneous tumor development. As both aging and cancer are associated with altered stem cell function, the possibility that the involvement of sirtuins in these events is mediated by their roles in stem cells is worthy of investigation. Research to date suggests that the individual sirtuin family members can differentially regulate embryonic, hematopoietic as well as other adult stem cells in a tissue‐ and cell type‐specific context. Sirtuin‐driven regulation of both cell differentiation and signaling pathways previously involved in stem cell maintenance has been described where downstream effectors involved determine the biological outcome. Similarly, diverse roles have been reported in cancer stem cells (CSCs), depending on the tissue of origin. This review highlights the current knowledge which places sirtuins at the intersection of stem cells, aging, and cancer. By outlining the plethora of stem cell‐related roles for individual sirtuins in various contexts, our purpose was to provide an indication of their significance in relation to cancer and aging, as well as to generate a clearer picture of their therapeutic potential. Finally, we propose future directions which will contribute to the better understanding of sirtuins, thereby further unraveling the full repertoire of sirtuin functions in both normal stem cells and CSCs.

## Introduction: Sirtuins

Sirtuins are members of the class III histone deacetylase family of enzymes that share a conserved 275‐amino acid catalytic core domain and are dependent on nicotinamide adenine dinucleotide (NAD^+^) for their activity (Vassilopoulos *et al*., 2011). Phylogenetic analysis divides the seven mammalian sirtuins (SIRT1‐7) into four classes: SIRT1‐3 are in class I, SIRT4 in class II, SIRT5 in class III, and SIRT6‐7 in class IV (Frye, 2000). Mammalian sirtuins may also be categorized according to their subcellular localization: SIRT1, 6, and 7 are present in the nucleus, SIRT3, 4, and 5 in the mitochondria, and SIRT2 is found predominately in the cytoplasm (Houtkooper *et al*., 2012). This diversity in subcellular location, combined with differing expression patterns and distinct substrates, contributes to the diverse biological functions of the individual family members. Although initially described as histone deacetylases in yeast, mammalian sirtuins also regulate an inestimable range of nonhistone cellular proteins through lysine deacetylation. The repertoire of sirtuin function has continued to expand since their discovery, with roles in additional PTMs being increasingly reported. In this regard, SIRT6 has been shown to regulate deacylation (Jiang *et al*., 2013) and, as well as SIRT4, can function as an ADP‐ribosyltransferase (Liszt *et al*., 2005; Haigis *et al*., 2006b). In addition, SIRT5 is capable of desuccinylation and demalonylation (Du *et al*., 2011; Park *et al*., 2013a).

In addition to subcellular localization and NAD^+^ availability, there are multiple additional mechanisms of regulation that contribute to sirtuin activity and specificity. This ensures activation of different sirtuins and consequent stimulation of distinct and diverse substrates. Transcriptional regulation including various transcription factors/repressors, miRNAs, post‐translational regulation, protein–protein interactions, and regulation by small molecules have all been described (reviewed in Houtkooper *et al*. (2012)). By employing such regulatory mechanisms, environmental stimuli including calorie restriction (CR) are known to control sirtuin expression and/or activity. Therefore, sirtuins are considered to be stress‐responsive enzymes that direct cellular adaptations by altering the acetylome. Although currently debated, sirtuins have been shown to regulate longevity in numerous lower organisms including yeast, nematodes, and fruit flies (Haigis & Guarente, 2006a; Burnett *et al*., 2011) as well as higher organisms such as mice (Kanfi *et al*., 2012). Consequently, mammalian sirtuin research has to date been intensely focused on their roles in aging and aging‐related diseases. As a result of the variety of proteins that can be regulated by lysine acetylation, sirtuins have been shown to be master regulators of diverse cellular activities such as gene expression, metabolism, telomere activity, cell cycle, differentiation, EMT, apoptosis, proliferation, DNA repair, senescence, and oxidative stress response. Interestingly, many of these are critical processes in the maintenance and differentiation of both normal stem cells and CSCs. In this review, we outline the roles various members of the sirtuin family play in some of these pathways and discuss the potential therapeutic implications of targeting sirtuins for the treatment of cancer and other stem cell‐related diseases.

## Sirtuins and stem cells

### Embryonic stem cells and development

Histone acetylation undergoes dynamic changes during differentiation of embryonic stem cells (ESCs) and appears to play an important role in development as a result. Particularly, ESCs display higher levels of histone acetylation than lineage‐restricted and more differentiated cells (Efroni *et al*., 2008). Thus, it is not surprising that sirtuins have been linked to development and differentiation of ESCs. It is important to note here that early embryonic development is reported to be normal in most sirtuin‐knockout mice; however, *Sirt1* knockout results in significant lethality during the fetal stage or soon after birth, with severe developmental defects (Cheng *et al*., 2003; Haigis *et al*., 2006b; Mostoslavsky *et al*., 2006; Lombard *et al*., 2007; Vakhrusheva *et al*., 2008; Du *et al*., 2011; Kim *et al*., 2011). As a result, SIRT1 is considered to be the most important sirtuin in these processes and is consequently the best studied in this context. As *Sirt1* is highly expressed in ESCs before being downregulated by miRNAs during differentiation (Saunders *et al*., 2010), it is thought to play a role in maintaining stemness of ESCs and appears to be involved in developmental programs upon differentiation of ESCs (Table 1). The role SIRT1 plays in ESC differentiation differs depending on environmental conditions – loss of *Sirt1* under normal conditions does not induce differentiation; however under oxidative stress, Sirt1 mediates the maintenance of stemness promoting mitochondrial over nuclear translocation of p53 and maintaining *Nanog* expression (Han *et al*., 2008; Calvanese *et al*., 2010). SIRT1 is a known component of Polycomb repressive complex 4 (PRC4), which represses developmental genes in ESCs (Kuzmichev *et al*., 2005) and also binds to the promoters of development‐associated genes in ESCs, such as *TBX3* and *PAX6* where it contributes to gene silencing. As a result of its ability to regulate stemness and pluripotency factors, the role of SIRT1 in cellular reprogramming of somatic cells to induced pluripotent stem cells (iPSCs) has also been investigated. Both *SIRT1* overexpression and treatment with the known sirtuin activator resveratrol have been shown to enhance the efficiency of iPSC generation, whereas *Sirt1* knockdown exerts opposite action. This effect is associated with deacetylation of p53 and increased *Nanog* expression (Lee *et al*., 2012).

**Table 1:** Sirtuin functions and mechanisms of action in stem cells

Sirtuin	Action	Mechanism	Cells/Tissue	References
SIRT1	Maintenance of stemness	Mitochondrial translocation of p53 maintains Nanog expression	ESC	Han *et al*. (2008)
SIRT1	Maintenance of stemness	Component of PRC4 represses developmental genes	ESC	Kuzmichev *et al*. (2005)
SIRT1	Maintenance of stemness	ROS elimination, FOXO activation, and inhibition of p53	HSC	Matsui *et al*. (2012)
SIRT1	Promotes differentiation	Interacts with N‐CoR to block Notch‐Hes1 signaling	NSC	Hisahara *et al*. (2008)
SIRT2	Promotes differentiation	Negatively regulates GSK3β	ESC	Si *et al*. (2013)
SIRT3	Maintenance of stemness	Required for HSC self‐renewal at old age, related to oxidative stress	HSC	Brown *et al*. (2013)
SIRT6	Promotes differentiation	Regulates acetylation of H3K56 and H3K9 at *Oct4* and *Sox2* promoters	ESC	Etchegaray *et al*. (2015)
SIRT6	Maintenance of stemness	Represses Wnt target genes by interacting with LEF1 and deacetylating histone 3	HSC	Wang *et al*. (2016)
SIRT7	Maintenance of stemness	Regulates UPR^mt^ and NRF1	HSC	Mohrin *et al*. (2015)

Although less comprehensively studied, other sirtuins have been implicated in the regulation of cell lineage specification during ESC differentiation (Table 1). *Sirt2* is upregulated during mouse ESC differentiation and negatively regulates glycogen synthase kinase‐3β (GSK3β), a negative regulator of the Wnt/β‐catenin pathway. It was found that *Sirt2* knockdown compromised differentiation of mouse ESCs into ectoderm while promoting mesoderm and endoderm differentiation (Si *et al*., 2013). Conversely, ESCs from *Sirt6*‐knockout mice display upregulation of ectoderm markers and downregulation of genes associated with endoderm and mesoderm, thus highlighting the sometimes opposing roles of sirtuin family members. SIRT6 controls ESC differentiation by regulating acetylation of H3K56 and H3K9 at the *Oct4* and *Sox2* promoters. By repressing expression of these pluripotency genes, SIRT6 diminishes the expression of *Tet* enzymes, limits the levels of 5hmC, and allows balanced transcription of developmentally regulated genes (Etchegaray *et al*., 2015).

### Hematopoietic stem cells

As the first family member to be discovered, SIRT1 is also the best studied in other types of stem cells. In particular, its role in hematopoietic stem cells (HSCs), where it is expressed in both human and mouse cells of all lineages and stages of maturation, is well understood. A number of *in vivo* studies that utilize *Sirt1* ^*−/−*^ mice have demonstrated that SIRT1 positively regulates stemness in HSCs (Table 1). In embryonic hematopoietic development, *Sirt1* ^*−/−*^ ESC formed fewer mature blast cell colonies, with defective hematopoietic potential associated with delayed deactivation of *Oct4*,* Nanog*, and *Fgf5* expression (Ou *et al*., 2011). Consistent with a role of SIRT1 in mouse hematopoiesis and differentiation, another study demonstrated that SIRT1 does contribute to the maintenance of the HSC pool as murine bone marrow c‐Kit^high^Sca‐1^+^Lineage^−^ cells isolated from *Sirt1* ^*−/−*^ mice more readily differentiate and lose stem cell characteristics than wild‐type HSC. The mechanism behind SIRT1 maintenance of hematopoietic cell stemness was found to involve ROS elimination, FOXO activation, and inhibition of p53 (Matsui *et al*., 2012). According to these previous findings, it would be expected that SIRT1 is indispensable for normal function of HSCs. However, a recent *in vivo* study showed that *Sirt1* deletion had no effect on the production of mature blood cells, lineage distribution within hematopoietic organs, and frequencies of the most primitive HSC populations (Leko *et al*., 2012). Specific hematopoietic cell‐knockout and inducible *Sirt1*‐knockout mouse models have also contributed to the understanding of SIRT1 function in HSCs while overcoming the experimental challenges related to the developmental defects and perinatal death of *Sirt1*‐knockout mice. Following tamoxifen‐induced *Sirt1* deletion, a gradual increase in the total number and the frequency of HSCs as well as an expansion of the myeloid lineage at the expense of lymphoid cells were observed (Rimmelé *et al*., 2014). As above, this study also identified FOXO3 as an important mediator of the homeostatic control by SIRT1 in HSCs. Results obtained regarding the role of SIRT1 in regulating HSCs under stressful conditions serve to highlight the significant role the extracellular context may play in directing sirtuin function in stem cells. In the fraction of *Sirt1* ^*−/−*^ mice that survive postnatally, loss of SIRT1 is associated with decreased hematopoietic progenitors particularly under hypoxic conditions (Ou *et al*., 2011). This is consistent with a recent study showing that deletion of SIRT1 specifically in hematopoietic cells, after crossing Sirt1‐floxed mice with vav‐iCre transgenic mice, promotes aberrant HSC expansion and exhaustion but only under conditions of hematopoietic stress (Singh *et al*., 2013).

A similar experimental *in vivo* approach has been followed to uncover the role of SIRT6 in HSCs (Table 1). Using *Sirt6* ^fl/fl^ Vav‐Cre mice for hematopoietic‐specific deletion, a pIpC‐inducible mouse model (*Sirt6* ^fl/fl^ Mx1‐Cre), and *Sirt6* ^fl/fl^ ERT2‐Cre mice for inducible deletion in adult HSCs, it has been shown that *Sirt6* deficiency results in a significant increase in the number of immunophenotypically defined HSCs (Wang *et al*., 2016). However, SIRT6‐deficient HSCs exhibited a remarkable decrease in the long‐term multilineage repopulating activity, which is similar to the previously described effect of *Sirt1* loss. The phenotypic expansion and functional decline of SIRT6‐deficient HSCs is associated with an abnormal hyperproliferation induced by aberrant activation of Wnt signaling pathway.

SIRT3 and SIRT7 are also involved in HSC maintenance through the regulation of mitochondrial homeostasis (Table 1). Although SIRT3 seems to be dispensable for HSC maintenance at a young age, *Sirt3* deficiency results in a reduced HSC pool at an old age and compromised HSC self‐renewal upon serial transplantation stress (Brown *et al*., 2013). These phenotypes are attributed to increased oxidative stress due to decreased antioxidant activity of acetylated MnSOD upon *Sirt3* loss. Interestingly, *Sirt7* genetic inactivation also results in compromised regenerative capacity of HSCs, in this instance by failing to alleviate mitochondrial protein folding stress. *Sirt7* ^−/−^ bone marrow cells or purified HSCs display a 40% reduction in long‐term reconstitution of the recipients’ hematopoietic system compared with their *Sirt7* ^+/+^ counterparts. Even though *Sirt7* loss does not affect HSC frequency in the bone marrow under steady‐state conditions, a 50% reduction in the frequency of *Sirt7* ^−/−^ HSCs upon transplantation is observed coupled with increased apoptosis (Mohrin *et al*., 2015).

## Cell differentiation

Given that the balance between cell differentiation and self‐renewal is critical for adult stem cell maintenance and tissue regeneration, studies have revealed a role for sirtuins as regulators of differentiation in several cell types. In normal differentiation of neural stem cells (NSCs), SIRT1 translocates to the nucleus where it interacts with the nuclear receptor corepressor (N‐CoR) to block Notch‐Hes1 signaling and promote neuronal differentiation (Hisahara *et al*., 2008) (Table 1). However, stress conditions appear to particularly affect SIRT1 functions in stem cell differentiation pathways, even within the same cell type. Therefore, mild oxidation causes SIRT1 to bind to Hes1 and directs NSC differentiation toward the astroglial lineage rather than neuronal (Prozorovski *et al*., 2008), which might facilitate astrogliosis and healing in response to brain and spinal cord injuries. In further support of its role in regulating differentiation pathways above, SIRT1 has been reported to be involved in muscle differentiation. Under fasting conditions, which are known to activate sirtuins, SIRT1 responds to the altered [NAD(+)]/[NADH] ratio to inhibit muscle differentiation through deacetylation of PCAF and MyoD (Fulco *et al*., 2003). SIRT1 has also been shown to suppress differentiation in iPSCs as well. During the generation of NSCs from mouse iPSCs, levels of *Sirt1* have been observed to decrease, whereas miRNA‐34a, an inhibitor of SIRT1, increases. Furthermore, pharmacologic inhibition of SIRT1 using nicotinamide (NAM) enhanced the generation of NSCs and mature nerve cells (Hu *et al*., 2014a). Although it is generally considered a SIRT1 suppressor, mi‐R34a positively regulates SIRT1 during smooth muscle cell (SMC) differentiation from pluripotent stem cells. In this cell‐specific context, SIRT1 positively regulates differentiation by promoting the expression of transcription factors that regulate SMC genes by inhibiting H3K9 methylation (Yu *et al*., 2015).

SIRT2 also plays complex roles in both promoting and suppressing differentiation depending on the tissue studied. SIRT2 may positively regulate differentiation of keratinocytes – loss of *Sirt2* is associated with increased expression of epidermal stem cell markers keratin‐5, keratin‐19, and CD34, as well as decreased expression of loricrin, a marker of terminal keratinocyte differentiation (Ming *et al*., 2014). Focusing on adipogenesis and consistent with a negative role described for SIRT1 in this process (Picard *et al*., 2004), SIRT2 has been shown to inhibit preadipocyte differentiation in 3T3‐L1 cells. Downregulation of *Sirt2* increases acetylation of FOXO1, thereby affecting FOXO1 phosphorylation, nuclear/cytoplasmic localization, and ultimately activity, resulting in adipogenesis (Jing *et al*., 2007).

Given that unique expression patterns and substrate specificity may dictate cellular functions regulated by the different members of the sirtuin family, SIRT3 appears to be required for the differentiation of brown adipocytes in contrast to the negative effect of both SIRT1 and SIRT2 in adipogenesis. The coordinated action of the transcriptional coactivator peroxisome proliferator‐activated receptor‐γ coactivator‐1α (PGC‐1α) with the orphan nuclear receptor estrogen‐related receptor‐α induces *Sirt3* gene expression in white adipocytes and embryonic fibroblasts. This seems to be required for the induction of a brown adipose tissue‐specific pattern of gene expression, as evidenced by the finding that PGC‐1α fails to fully induce brown adipose tissue‐specific gene expression in cells lacking *Sirt3* (Giralt *et al*., 2011).

## Sirtuins in stem cell signaling pathways

**Figure 1:**
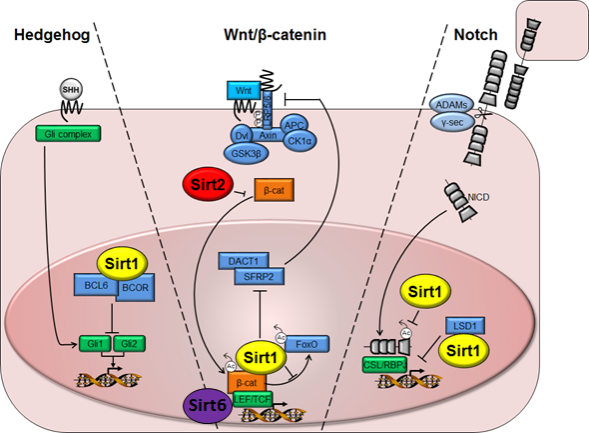
Schematic representation of sirtuin roles in Hedgehog, Wnt/β‐catenin, and Notch stem cell signaling pathways. By interacting with BCL6 and BCOR, Sirt1 represses Sonic Hedgehog effectors Gli1 and Gli2. Sirt1 promotes Wnt/β‐catenin signaling by deacetylating β‐catenin and FOXO transcription factors and by suppressing Wnt pathway antagonists SFRP2 and DACT1. Sirt2, however, inhibits β‐catenin signaling and downregulates expression of Wnt target genes, while Sirt6 represses Wnt target genes by interacting with LEF1 and deacetylating histone 3. With the focus on Notch pathway, Sirt1 deacetylates and destabilizes the NICD. Sirt1 also cooperates with LSD1 to repress Notch target genes. ADAMs, a disintegrin and metalloproteases; APC, adenomatous polyposis coli; CK1α, casein kinase 1α; CSL/RBPJ, CBF1 Suppressor of Hairless LAG‐1/recombination signal binding protein for immunoglobulin κ J region; Dvl, Disheveled; GSK3β, glycogen synthase kinase‐3β; LEF/TCF, lymphoid enhancer factor/T‐cell factor; LSD1, lysine demethylase 1A; LRP5/6, low‐density lipoprotein‐related proteins 5 and 6; NICD, Notch intracellular domain; SHH, Sonic Hedgehog; β‐cat, β‐catenin; γ‐sec, γ‐secretase.

Signaling pathways that regulate stem cell function are crucial for normal embryonic development and adult tissue homeostasis. Pathways such as Hedgehog, Wnt, and Notch, among others, are critical players in controlling the intricate balance of properties that define a stem cell, including self‐renewal and differentiation, even though their significance may vary in different stem cell populations. These pathways are strictly controlled by epigenetic regulation such as DNA methylation and histone modification (Toh *et al*., 2017). Furthermore, epigenetic changes in response to environmental signals, including nutrient stress, are known to alter stem cell pathway function (Brunet & Rando, 2017). It comes as no surprise therefore that sirtuins, particularly SIRT1, have been shown to interact with various components of these signaling networks (Fig. 1). Even though not all studies mentioned below that outline interactions between sirtuins and these pathways have been performed in stem cells, it is clear that members of the sirtuin family are actively involved in these signaling pathways with the notion that the underlying biology remains to be further explored in a stem cell‐specific context.

### Hedgehog

The Hedgehog pathway controls normal development and organ patterning during embryogenesis and maintains adult tissue homeostasis by controlling cellular proliferation and differentiation (Matsui, 2016). Upon Sonic Hedgehog (Shh) binding to the Patched receptor, the transmembrane protein Smoothened is activated and allows the release and nuclear translocation of Gli transcription factors to mediate expression of Hedgehog target genes. SIRT1 has been identified *in vivo* as a negative regulator of this pathway in neuron precursors. In complex with BCL6/BCOR, SIRT1 epigenetically represses Shh signaling effectors Gli1 and Gli2, which are responsible for the expression of genes required for normal cerebellar development. Of note, this same mechanism of epigenetic regulation also acts as a tumor suppressor in medulloblastoma where the Hedgehog pathway is activated by genetic mutations. This suggests that activation of the BCL6/BCOR/SIRT1 complex may be exploited therapeutically in Sonic Hedgehog‐dependent tumors (Tiberi *et al*., 2014). Although not reported in a stem cell context, SIRT6 appears to regulate expression of another Hedgehog ligand, Indian hedgehog (*Ihh*), and its downstream genes. Chondrocytes from *Sirt6* ^*−/−*^ mice exhibit decreased expression of *Ihh*, impaired proliferation and differentiation, and a senescent phenotype (Piao *et al*., 2013).

### Wnt

The canonical Wnt pathway activates gene transcription via β‐catenin. Upon Wnt ligand binding to Frizzled and/or lipoprotein‐related (LRP) 5 and 6 coreceptors, the degradation complex that inactivates β‐catenin is disassembled, allowing stabilization and nuclear translocation of accumulated β‐catenin. The canonical Wnt pathway regulates proliferation and survival and alterations in epigenetic control of the pathway are associated with a variety of cancers (Toh *et al*., 2017). Increasing evidence suggests that SIRT1 promotes Wnt/β‐catenin signaling in both normal progenitor and cancer cells by a variety of mechanisms. These include both activation of essential pathway components, including β‐catenin itself, and inhibition of Wnt pathway antagonists. SIRT1 is known to deacetylate β‐catenin. This promotes nuclear accumulation of β‐catenin and the transcription of Wnt/β‐catenin target genes in both adipogenesis and osteogenesis (Feng *et al*., 2016; Zhou *et al*., 2016b). In osteoblast progenitors, deacetylation of FOXO transcription factors by SIRT1 promotes Wnt signaling and bone formation by preventing sequestration of β‐catenin by FOXOs (Iyer *et al*., 2014). In mesenchymal stem cells, SIRT1 activates Wnt/β‐catenin signaling and promotes myogenic differentiation by suppressing expression of the Wnt pathway antagonists SFRP2 and DACT1 (Zhou *et al*., 2015). Like SIRT1, SIRT2 has been shown to bind directly to β‐catenin. However while SIRT1 activates β‐catenin, SIRT2 has been shown to inhibit the Wnt signaling pathway, again highlighting the sometimes opposing actions of different members of the sirtuin family. SIRT2 binding to β‐catenin occurs particularly in response to oxidative stress by ionizing radiation. This interaction impairs expression of Wnt target genes such as survivin, cyclin D1, and c‐myc (Nguyen *et al*., 2014). SIRT6 also inhibits transcription of Wnt target genes. Unlike SIRT2, repression is not a result of interaction between SIRT6 and Wnt but rather direct binding of SIRT6 with the transcription factor LEF1 and deacetylation of histone 3. Notably, this mechanism of epigenetic regulation of Wnt signaling by SIRT6 was described in HSCs *in vivo* and plays a role in HSC homeostasis whereby SIRT6 is required to maintain HSC self‐renewal ability as discussed above (Wang *et al*., 2016).

### Notch

Notch ligands and receptors are transmembrane proteins that mediate cell contact‐dependent signaling. Ligand binding causes cleavage and nuclear translocation of the Notch intracellular domain (NICD) where it activates transcription of target genes. Notch signaling activity is known to be regulated by epigenetic control of several components of the Notch pathway, including Notch itself. Although a role for sirtuins in Notch signaling in stem cells has not been described, SIRT1 in particular is capable of regulating the activity of components of the Notch pathway. In fact, SIRT1 appears to be both a direct and indirect negative regulator of Notch signaling, particularly in endothelial cells where research has focused to date. SIRT1 directly regulates Notch1 by deacetylating conserved lysines in the NICD. This destabilizes Notch1 and has been shown to limit Notch signaling in endothelial cells (Guarani *et al*., 2011). SIRT1 also inhibits Notch pathway signaling indirectly via its H4K16 deacetylation activity. SIRT1 and lysine demethylase 1A (LSD1) interact directly and play conserved and concerted roles in H4K16 deacetylation and H3K4 demethylation to repress Notch target genes (Mulligan *et al*., 2011).

Other members of the sirtuin family have been found to regulate Notch signaling pathways in the context of cancer cells. SIRT3 suppresses both mRNA and protein expression of NOTCH1 in gastric cancer cells. This inhibition was associated with decreased proliferation and colony formation (Wang *et al*., 2015). Similarly, SIRT6 has been shown to inhibit proliferation of ovarian cancer cells by downregulating *NOTCH3* mRNA and protein levels (Zhang *et al*., 2015).

## Sirtuins and cancer stem cells

The stem cell signaling pathways discussed above are often aberrantly activated or suppressed in cancer due to deregulation of epigenetic control. As a result, sirtuins may be implicated in the generation of a population of cancer cells capable of self‐renewal and differentiation which drive tumor growth. Evidence also suggests that, at least in some tissues, distinct stem cell programs exist in CSCs and normal stem cells of the corresponding tissue (Ye *et al*., 2015). As a result, sirtuins may play unique roles in CSCs in addition to those observed in normal stem cells.

As in normal stem cells, SIRT1 is the best studied sirtuin in CSCs. *SIRT1* expression has been shown to be upregulated in a variety of CSCs both *in vitro* and *in vivo*, including glioma (Lee *et al*., 2015), breast (Ma *et al*., 2015), colorectal (Chen *et al*., 2014b), and leukemia (Li *et al*., 2012, 2014). SIRT1 is required for both oncogenic transformation and maintenance of stemness in glioma cells (Lee *et al*., 2015). Consequently, CD133^+^ glioma stem cells express high levels of SIRT1 compared to CD133^‐^ non‐stem cells. Notably, knockdown of *SIRT1* increased the radiosensitivity of CD133^+^ cells both *in vitro* and *in vivo* (Chang *et al*., 2009). High levels of SIRT1, as well as low expression of its direct regulator miR‐34a, have been identified in CD44^+^/CD24^‐^ breast cancer stem cells (BCSCs). Both overexpression of miR‐34a and knockdown of *SIRT1* were found to decrease BCSCs, tumorsphere formation, and expression of CSC markers including ALDH1 and Nanog (Ma *et al*., 2015). In colorectal cancer cells, SIRT1 is also overexpressed and colocalizes with the colorectal CSC marker CD133. Knockdown of *SIRT1* reduced CD133^+^ cells, decreased sphere formation, and attenuated tumorigenicity *in vivo*. Furthermore, the expression of several stem cell markers, including *OCT4*,* NANOG*, and *TERT* was also found to be decreased (Chen *et al*., 2014b). *SIRT1* is highly expressed in Nanog^POS^ liver CSCs but decreases during differentiation. Consequently, it has been shown to be responsible *in vitro* for the maintenance of self‐renewal in liver CSCs by epigenetic regulation of the *SOX2* promoter (Liu *et al*., 2016). It is clear therefore that SIRT1 is required for the maintenance of CSCs. Although the precise downstream mechanisms involved may vary, loss of SIRT1 is associated with decreased sphere formation, reduced expression of CSC markers, and increased sensitivity to treatment. Outside of its specific roles identified in CSCs, SIRT1 is also known to regulate the stemness‐associated Wnt signaling pathway in several non‐stem cancer cell contexts. It mediates epigenetic silencing of Wnt antagonists SFRP1, SFRP2, and DKK1 in addition to positively regulating levels of all three mammalian Disheveled (Dvl) proteins (Pruitt *et al*., 2006; Hussain *et al*., 2009; Holloway *et al*., 2010). In breast cancer cells, inhibition of SIRT1/2 decreased Frizzled7 (FZD7) protein expression, as well as β‐catenin and c‐Jun binding to the *FZD7* promoter (Simmons *et al*., 2014). Similarly, inhibition of SIRT1 in acute lymphoblastic leukemia (ALL) also inhibits the Wnt/β‐catenin signaling pathway resulting in elimination of ALL stem/progenitor cells (Jin *et al*., 2015).

In addition to the regulatory role of sirtuins in distinct stem cell programs, it has been shown that they may directly regulate the activity of CSC markers themselves. In this regard, ALDH1A1 activity is a commonly used marker for CSCs and evidence suggests that it may be involved in CSC maintenance or differentiation. SIRT2 has recently been shown to post‐translationally regulate ALDH1A1. NOTCH signaling induces SIRT2 to deacetylate ALDH1A1, leading to increased ALDH activity, CSC populations, and CSC self‐renewal in breast cancer (Zhao *et al*., 2014). On the other hand, SIRT2 activity has been reported to exert an opposite effect in the context of glioblastoma. There, SIRT2 activation mediates the antiproliferative function of resveratrol specifically on glioblastoma stem cells (Sayd *et al*., 2014), thus underlining the importance of cellular context in determining specific roles for sirtuins.

Consistent with the previously described tumor suppressive role for SIRT6, its overexpression results in suppressed CSC activity in breast, lung, and colorectal cancer cells with PI3K activation as evidenced by the decreased tumorsphere‐forming capacity and size of ALDH^+^ cell population which are established readouts of CSCs (Ioris *et al*., 2017). These results were further confirmed *in vivo* after cell injection into the flanks of nonobese diabetic/severe combined immunodeficient (NOD/SCID) mice as well as after monitoring tumor growth in transgenic mice expressing polyomavirus middle T oncogene (PyMT) under the mouse mammary tumor virus promoter. In both cases, SIRT6 overexpression delays tumor growth, which is associated with decreased ALDH expression and enzymatic activity, altered glucose and lipid metabolism, and reduced cancer stemness. From a mechanistic point of view, enhanced SIRT6 dampens phosphoinositide 3‐kinase (PI3K) signaling at the transcriptional level counteracting the positive role of PI3K signaling in survival and maintenance of CSCs.

### Epithelial–Mesenchymal Transition (EMT)

**Figure 2:**
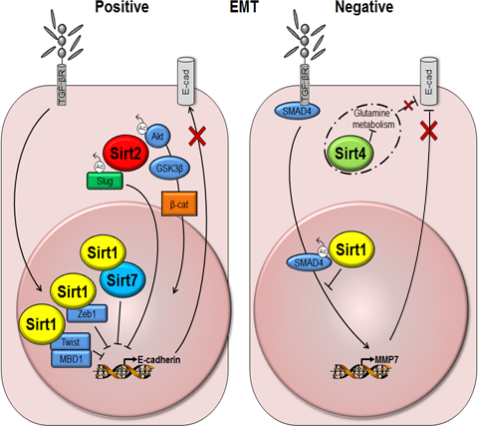
Schematic representation of positive and negative regulation of EMT by sirtuins. Positive: TGF‐β signaling is associated with an increase in Sirt1. Sirt1 is recruited by Zeb1 to the E‐cadherin promoter and causes transcriptional repression. Similarly, Sirt1 interacts with Twist and MBD1 to silence the E‐cadherin promoter. Sirt1 may also recruit Sirt7 to repress E‐cadherin expression. Sirt2 activates Akt/GSK3β/β‐catenin signaling to promote EMT. Deacetylation of Slug by Sirt2 promotes Slug protein stability and repression of Slug target genes, including E‐cadherin. Negative: Sirt1 inhibits TGF‐β signaling by deacetylating Smad4. This decreases MMP transcription and E‐cadherin degradation. Sirt4 upregulates E‐cadherin expression via its repression of glutamine metabolism. E‐cad, E‐cadherin; GSK3β, glycogen synthase kinase‐3β; MBD1, methyl‐CpG binding domain protein‐1; MMP7, metalloproteinase 7; TGF‐βR, transforming growth factor‐β receptor; β‐cat, β‐catenin.

The EMT program consists of multiple transitional states in which cells move through between epithelial and mesenchymal phenotypes (Nieto *et al*., 2016). During EMT, epithelial cells reorganize their cytoskeleton, lose apical–basal polarity and cell–cell adhesion, and attain an increased capacity for cell mobility. Importantly, activation of EMT programs confers stem‐like traits on both normal and neoplastic cells (Mani *et al*., 2008). Several reports have outlined roles for various sirtuins in the promotion of EMT. However, consistent with their characterization as either tumor promoters or suppressors depending on cellular context, sirtuins have also been described as both enhancers and repressors of EMT (Fig. 2). The actual functions then may differ between individual sirtuins, tissues of origin, microenvironments, and cellular contexts. Furthermore, the biochemical mechanisms that underpin sirtuin regulation of EMT remain unclear and it is not yet known whether all of these effects involve deacetylase activity.

SIRT1 has been shown to be a positive regulator of EMT in prostate cancer, through its deacetylase activity. SIRT1 is recruited to the E‐cadherin promoter by the zinc finger transcription factor ZEB1. Here, it deacetylates histone H3, reduces binding of RNA polymerase II ultimately causing transcriptional repression of E‐cadherin. As a result, loss of *SIRT1* decreases prostate cancer cell migration and metastasis, independent of any effects on cell survival (Byles *et al*., 2012). Similarly, SIRT1 interacts with Twist and methyl‐CpG binding domain protein‐1 (MBD1) to silence the E‐cadherin promoter in pancreatic cancer (Xu *et al*., 2013). Furthermore, EMT in pancreatic cancer cells, induced by transforming growth factor‐β (TGF‐β), is associated with upregulation of *SIRT1*, while inhibition of SIRT1 induced mesenchymal–epithelial transition (Deng *et al*., 2014). On the other hand, deacetylation of Smad4 by SIRT1 was identified as a mechanism of reducing EMT by repressing the effects of the TGF‐β signaling pathway in both transformed primary human mammary epithelial cells and kidney epithelial cells (Simic *et al*., 2013). Moreover, SIRT1 has been identified as an EMT repressor in oral squamous cell carcinoma, lung cancer, and ovarian cancer (Sun *et al*., 2013a,b; Chen *et al*., 2014a).

Despite the reduced expression of E‐cadherin observed in SIRT2^*−/−*^ MEFs (Nguyen *et al*., 2014), SIRT2 has been shown to positively regulate EMT in the context of cancer. SIRT2 expression is upregulated in hepatocellular carcinoma where it promotes EMT by deacetylating and activating protein kinase B to target the Akt/GSK3β/β‐catenin signaling pathway (Chen *et al*., 2013). Consistent with its positive role, a recently published study showed that SIRT2 maintains Slug protein levels through deacetylation‐mediated increased protein stability. Furthermore, it was shown that elevated Slug protein caused by SIRT2 overexpression corresponded to stronger repression of the Slug transcriptional targets, epithelial cell adhesion molecule, and E‐cadherin, implying that SIRT2 might regulate EMT‐related phenotypes such as aggressiveness and invasion specifically in triple‐negative basal‐like breast cancer (Zhou *et al*., 2016a).

With emphasis on the diverse functions regulated by the different members of the sirtuin family, SIRT4 has been described as a negative regulator of EMT. *SIRT4* expression is associated with upregulation of E‐cadherin expression and decreased vimentin expression in colorectal cancer cells. Under these experimental conditions, SIRT4 suppresses glutamine metabolism by repressing the enzymatic activity of glutamate dehydrogenase. Given that α‐ketoglutarate, an important product of glutamine metabolism, inhibited the upregulation of E‐cadherin expression by SIRT4, it was suggested that regulation of E‐cadherin expression occurs via inhibition of glutamine metabolism (Miyo *et al*., 2015).

*SIRT7* expression has been found to correlate inversely with E‐cadherin in prostate cancer. Furthermore, loss of *SIRT7* expression in prostate carcinoma cell lines caused a reversal of the EMT phenotype with upregulation of E‐cadherin and decreased vimentin and Slug expression. Interestingly, it has been suggested that SIRT1 mediates the recruitment of SIRT7 to the E‐cadherin promoter and that this interplay between SIRT1 and ‐7 is responsible for promoting EMT (Malik *et al*., 2015). Additionally, overexpression of *SIRT7* in colorectal cancer is associated with downregulation of epithelial markers, including E‐cadherin, and upregulated expression of mesenchymal markers (Yu *et al*., 2014).

### Sirtuins as therapeutic targets for CSCs

Given that sirtuins can act as both cancer promoters and suppressors, both activators and inhibitors of sirtuins have been developed in recent years. Interestingly, inhibition of sirtuins has attracted more interest as a potential therapeutic anticancer strategy. To date, several sirtuin inhibitors have been developed which differ based on their mechanism of action and structural features (Hu *et al*., 2014b). Two classes of sirtuin inhibitors, NAM and thioacyllysine‐containing compounds, can be considered as mechanism‐based inhibitors, whereas other sirtuin inhibitors, including sirtinol and its analogues, splitomicin and its derivatives, indole derivatives as well as tenovin and its analogues, presumably work by noncovalent binding to the sirtuin active site and blocking substrate binding. With regard to anticancer effects, sirtuin inhibition has been found to induce growth arrest or cell death in various cancer cell lines from a wide range of tissues (Table 2) (Heltweg *et al*., 2006; Ota *et al*., 2006; Lara *et al*., 2009; Rotili *et al*., 2012b). Importantly, decreasing sirtuin activity has been shown to be effective in specifically targeting CSCs that are resistant to standard therapy. Tenovin‐6 (a small‐molecule inhibitor of SIRT1 and SIRT2) treatment yielded a significant loss of imatinib‐resistant chronic myeloid leukemia (CML) CD34+ stem cells *in vivo*. This effect was caused mainly by SIRT1 inhibition resulting in elevated acetylated and total p53 levels (Li *et al*., 2012). Similarly, it has been shown to eliminate CD133+ ALL stem cells and also decrease ALDH^+^ cells and tumorsphere formation in uveal melanoma cell lines (Jin *et al*., 2015; Dai *et al*., 2016). Furthermore, benzodeazaoxaflavin SIRT1/2 inhibitors have demonstrated antiproliferative activity in spheroidal cell cultures from both colon carcinoma and glioblastoma multiforme (Rotili *et al*., 2012a). In addition to antiproliferative potency against leukemia and breast cancer cell lines, the SIRT1/2 inhibitor salermide reduces viability in spheroidal cultures of colorectal CSC cell lines (Rotili *et al*., 2012b). In the same study, the SIRT2‐selective inhibitor AGK2 displayed antiproliferative activity against glioblastoma multiforme tumorspheres. It is worth noting, though, that in accordance with the previously reported opposing roles played by the different members of the sirtuin family and the undeniable significance of biological and contextual factors, a recent study suggested that sirtuin activation, rather than inhibition, could be a therapeutic strategy to target CSCs with activating PI3K mutations. More specifically, SIRT6 overexpression suppresses PI3K signaling at the transcriptional level and antagonizes tumorsphere formation. This implies that SIRT6 activation may be exploited therapeutically to hinder stemness of tumors with *PIK3CA* gene mutations or *PTEN* loss (Ioris *et al*., 2017).

**Table 2:** Sirtuin inhibitors with anticancer activity

Inhibitors	Target	Anti‐CSC activity
Tenovin‐6	SIRT1/2	CML, AML
BDF4‐1, ‐2a, ‐2b, ‐2d	SIRT1/2	Colon, glioblastoma
AGK2	SIRT2	Glioblastoma
Salermide	SIRT1/2	Colorectal
Inhibitors	Target	Anticancer activity
Nicotinamide	SIRT1/2/3/5/6	Leukemia, oral, prostate
Sirtinol	SIRT2	Breast, lung, prostate, oral
Salermide	SIRT1/2	Lung, breast, colon
JGB‐1741	SIRT1	Breast
Cambinol	SIRT1/2/5	Burkitt lymphoma
EX527	SIRT1	Leukemia
AC‐93253	SIRT1/2/3	Prostate, pancreas, lung
Inauhzin	SIRT1	Lung, colon
Tenovin‐1	Unknown	Burkitt lymphoma, melanoma

The upper part shows inhibitors that have been reported to exert an anti‐CSC‐specific effect (Li *et al*., 2012; Rotili *et al*., 2012a,b; Hu *et al*., 2014b; Jin *et al*., 2015; Dai *et al*., 2016).

## Sirtuins, aging, and stem cells

Sirtuins have long been recognized as regulators of aging – overexpression of sirtuins has been shown to extend lifespan in several organisms (Tissenbaum & Guarente, 2001; Kanfi *et al*., 2012). Sirtuin function in aging has to date been reported to be related to their roles in regulation of energy metabolism, response to calorie restriction (CR), control of cell death, and circadian rhythms (Araki *et al*., 2004; Chang & Guarente, 2013; Guarente, 2013). A new mechanism of lifespan modulation by sirtuins has been gaining attention, related to their potential roles in cellular and mitochondrial protein homeostasis networks. Recent developments have highlighted the close relationship between healthy aging and protein homeostasis, or proteostasis (Kaushik & Cuervo, 2015; Walther *et al*., 2015). A gradual loss of proteostasis is associated with age (Labbadia & Morimoto, 2015) and the longest living organisms are known to have more stable proteasomes and active proteostasis networks (Perez *et al*., 2009; Treaster *et al*., 2014). Most importantly, enhancing the functionality of proteostasis networks has been shown to extend both lifespan and healthspan of certain organisms (Morimoto & Cuervo, 2014; Vilchez *et al*., 2014a; Labbadia & Morimoto, 2015). Given that sirtuins are well‐known lifespan modulators whose deficiencies have been linked to a higher incidence of age‐related diseases, the investigation of their roles in proteostasis networks would appear to be warranted. In fact, a relationship between sirtuins and ER stress appears to be conserved from C. elegans to mammals, indicating a crucial link between sirtuins and proteostasis (Viswanathan *et al*., 2005). SIRT1 is a known negative regulator of ER stress responses through deacetylating IRE‐1‐generated active XBP1 and subsequent inhibition of its transcriptional activity to promote ER stress‐induced apoptosis (Wang *et al*., 2011). SIRT1 also suppresses pERK‐eIF2α‐dependent translational inhibition (Ghosh *et al*., 2011). In breast cancer cells, the unfolded protein response (UPR) triggered by the accumulation of misfolded proteins in the mitochondria (UPR^mt^) requires the activation of SIRT3 together with CHOP and estrogen receptor alpha (ERα). By orchestrating both the antioxidant machinery and mitophagy in a CHOP‐ and ERα‐independent manner, SIRT3 contributes to overcoming proteotoxic stress and mitochondrial stress, which may represent an essential mechanism of adaptation of cancer cells (Papa & Germain, 2014).

Looking more closely into stem cells, they seem to have increased mechanisms to protect their proteasomes, and proteostasis networks impact their function (Vilchez *et al*., 2014b). This would appear to be related to the stem cell theory of aging, which suggests that a progressive decline in the self‐renewal of adult stem cells and their potential to differentiate into specific cell types in order to replenish the tissues of an organism underlie the mechanistic basis for aging. Although the age‐dependent loss of function of different types of adult stem cells has been reported, we are just now starting to understand the molecular mechanisms involved in this process. With the focus on sirtuins, SIRT7‐mediated alleviation of mitochondrial protein folding stress plays a critical role in modulating the aging process by regulating HSC quiescence and tissue maintenance (Mohrin *et al*., 2015). SIRT7 functions as a stress sensor in proliferating, metabolically active HSCs and reduces the expression of the mitochondrial translation machinery through repressing activity of the master regulator of mitochondria, nuclear respiratory factor 1 (NRF1), which is necessary to alleviate mitochondrial protein folding stress. Of note, rescue of the impaired reconstitution capacity in aged HSCs upon SIRT7 overexpression or NRF1 inactivation underscores the significance of sirtuin‐regulated proteostasis in maintaining stemness. Interestingly, decreased *Sirt3* expression in aged HSCs is associated with a concomitant repression of mitochondrial protective programs (Brown *et al*., 2013), which might result in compromised function of the previously described SIRT3‐directed UPR^mt^ pathway. Certainly, further studies need to address whether similar mechanisms are involved in other adult stem cells and tissues. Also, it would be interesting to see whether these mechanisms are crucial for self‐renewal and differentiation of CSCs based on the fact that CSCs resemble a proliferating, metabolically active normal stem cell. Furthermore, even though SIRT7 and SIRT3 cross at mitochondrial regulation, they do activate these protective mechanisms through their function in nucleus and mitochondria, respectively. As similar protective programs might be orchestrated by other sirtuins, it remains to be determined whether SIRT2, which is the main cytoplasmic sirtuin strongly downregulated in aged HSCs as well (Chambers *et al*., 2007), is involved in stem cell maintenance, and possibly, new pathways crucial for stem cell maintenance remain to be identified.

### Calorie restriction and stem cells

CR is one of the most potent dietary interventions for increasing lifespan and delays the onset of age‐related diseases including cancer (Wanagat *et al*., 1999; Longo & Fontana, 2010; Colman *et al*., 2014). It is accepted that its beneficial effects might relate, at least in some significant part, to epigenetically reprogramming stemness while prolonging the capacity of stem‐like cell states to proliferate, differentiate, and replace mature cells in adult aging tissues. This is based on studies showing that CR may maintain stem cell function of HSCs (Ertl *et al*., 2008), enhance stem cell availability and activity in the muscle of young and old animals (Cerletti *et al*., 2012), and increase hippocampal neural stem and progenitor cell proliferation in aging mice (Park *et al*., 2013b). Considering that sirtuins are NAD‐dependent protein deacetylases directly activated by CR, it could be proposed that they may mediate some of the beneficial effects of CR on normal stem cells in adult somatic tissues. However, this is a relatively unexplored area of research and there is lack of experimental evidence to either support or counter this hypothesis. Only very recently, it was reported that SIRT1 is necessary for the expansion of intestinal stem cells (ISCs) upon CR. More specifically, CR results in deacetylation of p70 ribosomal S6 kinase due to SIRT1 activation, which consequently promotes its phosphorylation by mammalian target of rapamycin complex 1 (mTORC1). This signal‐response mechanism mediates the increase in both protein synthesis and number of ISCs, even as mTOR signaling is turned down by CR in more differentiated cells (Igarashi & Guarente, 2016). Similarly, little is known about the role of sirtuins as mediators of the CR‐induced effect on tumorigenesis. As previously mentioned, cancer was among the age‐related diseases which exhibited a delayed onset in response to CR in several early studies (Hursting *et al*., 1994; Berrigan *et al*., 2002; Mai *et al*., 2003). Thus, it is rather surprising that there is a lack of experimental data to address the contribution of sirtuins in the inhibitory effect of CR on tumorigenesis. Regarding SIRT1, which is the only sirtuin studied so far, its overexpression failed to influence the anticancer effects of every‐other‐day fasting (a variation in CR), suggesting that SIRT1 may play a limited role in the effects of CR on cancer (Herranz *et al*., 2011). Undoubtedly, future studies are necessary to check more thoroughly the role of sirtuins under this setting. However, it seems a very intriguing question to ask whether sirtuin‐directed functions may regulate either CSCs or non‐CSCs, given that cancer is now viewed as a stem cell disease. This is further supported by recent evidence highlighting the effect of CR on unique characteristics of CSCs such as EMT (Dunlap *et al*., 2012), protein synthesis (Lamb *et al*., 2015), metabolic plasticity (Peiris‐Pages *et al*., 2016), as well as the importance of the HIF pathway in regulating metabolism, cellular responses to hypoxia and stemness (Lim *et al*., 2010; Zhong *et al*., 2010; Yun & Lin, 2014), which are all processes previously shown to be regulated by sirtuins. It is hoped that future research will shed light on mechanisms underlying the interplay between CR, sirtuins, and stem cells.

## Conclusion/Future directions

Emerging evidence suggests that sirtuins could be placed at the crossroads of stemness, aging, and cancer. This is based on the plethora of functions they regulate both in normal stem cells and in CSCs. However, it is clear that we are just starting to appreciate the importance of identifying specific processes regulated by the different members of the sirtuin family in a tissue‐, cell type‐, and genetic‐specific context. This might be necessary in order to gain a better understanding of their role and fill current knowledge gaps in the field. With this in mind, it is worth mentioning that most of the previous studies, including the published papers presented in this review article, have followed a targeted approach regarding elucidation of mechanisms regulated by sirtuins. To do so, they were focused on either unraveling how sirtuins regulate signaling pathways/processes already implicated in stemness or exploring whether previously well‐established functions of sirtuins play a significant role in stem cells. Toward this direction, it could be proposed that implementation of unbiased high‐throughput experimental approaches would provide more mechanistic insights. Proteomics have been employed in the past to identify sirtuin‐specific interacting proteins and substrates. The regulatory role of SIRT2 on anaphase‐promoting complex (APC/C) during mitosis was identified based on a proteomics approach that revealed its interaction with proteins of the complex including the APC activator proteins Cdc20 and Cdh1 (Kim *et al*., 2011). Recently, proteomics were used to elucidate the mitochondrial sirtuin protein interaction landscape showing that this experimental approach can uncover novel functions and/or substrates (Yang *et al*., 2016). Thus, it could be suggested that similar approaches on stem and progenitor cells or CSCs would identify novel functions of sirtuins. Furthermore, recent advances in high‐resolution mass spectrometry‐based proteomics have enabled the study of the acetylome under different experimental conditions establishing acetylation as an equally widespread PTM as phosphorylation (Kim *et al*., 2006; Choudhary *et al*., 2009, 2014). Given that similar approaches have enabled the identification of sirtuin‐specific deacetylation targets (Hebert *et al*., 2013; Vassilopoulos *et al*., 2014), it would be reasonable to suggest that studying the acetylome in the context of stem cells/progenitors or CSCs would reveal novel functions/substrates regulated at the post‐translational level. In a similar way, a detailed characterization of target genes epigenetically regulated by sirtuins in specific subcellular populations could help shape new directions in this field and complement previous comprehensive studies focused on the analysis of the transcriptome, DNA methylome, and histone modifications (Sun *et al*., 2014) in stem cells. Collectively, such studies will provide novel insights into both aging and cancer.

## Funding

A. Vassilopoulos was supported by R01CA182506‐01A1 and the Lynn Sage Foundation.

## Conflict of interest

The authors have no conflict of interests to declare.
